# Evaluation of a Liquid Media MALDI-TOF MS Protocol for the Identification of Dermatophytes Isolated from *Tinea capitis* Infections

**DOI:** 10.3390/jof8121248

**Published:** 2022-11-26

**Authors:** Pauline Lecerf, Roelke De Paepe, Yasaman Jazaeri, Anne-Cécile Normand, Delphine Martiny, Ann Packeu

**Affiliations:** 1 Dermatology Department, University Hospital Brugmann & Saint-Pierre, Université Libre de Bruxelles, 1020 Brussels, Belgium; 2 Mycology and Aerobiology Department, Sciensano, 1050 Brussels, Belgium; 3 Parasitology/Mycology Department AP-HP, Hôpitaux de Paris, 75013 Paris, France; 4 Department of Microbiology, Laboratoire Hospitalier Universitaire de Bruxelles Universitair Laboratorium Brussel (LHUB-ULB), Université Libre de Bruxelles (ULB), 1000 Brussels, Belgium; 5 Faculty of Medicine and Franco, University of Mons (UMONS), 7000 Mons, Belgium; 6 BCCM/IHEM Fungal Collection, Sciensano, Mycology and Aerobiology Section, 1050 Brussels, Belgium

**Keywords:** fungus, dermatophytes, *tinea capitis*, MALDI-TOF MS, in-house library, liquid culture

## Abstract

One of the most common types of tinea is the superficial infection of the hair and scalp area known as tinea capitis. It is responsible for frequent outbreaks in nurseries and schools and represents a global health problem. Correct identification of the infection agent is essential in the determination of the infection source, epidemiological course, and treatment initiation. The conventional identification methods (direct exam, culture, DNA sequencing) are time-consuming, require experienced staff, are time-consuming, and the latter is expensive for routine identifications. Matrix-assisted laser desorption ionization time-of-flight mass spectrometry (MALDI-TOF MS) is gaining new ground for routine identification of filamentous fungi. The main advantages of MALDI-TOF MS are its rapid and accurate identification capability, relatively low cost, and easy integration into the laboratory routine. Its accuracy heavily depends on the quality of the reference spectra database. Identification of clinical isolates with MALDI-TOF MS protocol requires a sub-culturing step to ensure reliable identification. It can take days to weeks before fungal growth appears on solid medium. In this study, a unique MALDI-TOF MS protocol using liquid cultures of dermatophyte species was developed in order to shorten the turnaround time for the culture and identification of clinical isolates. Material and Method A standard MALDI-TOF MS protocol was adapted for liquid instead of solid cultures. Three different databases were tested. Results Using the liquid media MALDI-TOF MS protocol, a global rate of 62% correct identification (RCI) was obtained, compared with 87% for the protocol based on solid cultures. Trichophyton tonsurans was not correctly identified in all isolates using liquid cultures, with 88% of the isolates misidentified as Trichophyton interdigitale. The turnaround time for primary isolates for the solid and liquid protocols were respectively 11.7 and 11.6 days (no significant difference between both methods (*p* = 0.96)). Conclusions The newly designed liquid MALDI-TOF MS protocol did not lead to a significantly shorter turnaround time for the identification of dermatophytes isolated from tinea capitis infections. The turnaround time for the method with primary isolates was not significantly lower, and the rate of correct identification decreased remarkably, which emphasizes the need for a sub-culturing step. Using different database did not lead to improvement in turnaround time or rate of correct identification. This study highlights the importance of the medium and the reference database when performing MALDI-TOF MS.

## 1. Introduction

The dermatophytes (*Arthrodermatacea* family) are a group of filamentous fungi that can invade and infect keratinized tissues [1]. Nine distinguished dermatophyte genera have been defined: *Arthroderma*, *Ctenomyces*, *Epidermophyton (E)*, *Guarromyces*, *Lophophyton*, *Microsporum (M)*, *Nannizzia (N)*, *Paraphyton*, and *Trichophyton (T)* [2]. Dermatophytes can be classified according to their natural habitats, resulting in groups of anthropophilic, zoophilic, and geophilic species. It is estimated that 20–25% of the world’s population suffers from skin mycoses. One of the most common types of tinea is the superficial infection of the hair and scalp area, known as tinea capitis. It is responsible for frequent outbreaks in nurseries and schools and represents a global health problem [1,3,4]. Tinea capitis is most prevalent in children, mainly those aged three to seven, with males more frequently affected than females [5,6,7]. Alopecic patches, broken hairs, scales, pustules, kerion, and possible regional lymphadenopathy are typical clinical manifestations [8,9,10]. Atypical clinical presentations with mild, nonspecific, or non-inflammatory signs make clinical diagnosis challenging (e.g.: appearance of seborrheic dermatitis with mild inflammation and scarce scaling). The main pathogens causing tinea capitis are species of the *Trichophyton* and *Microsporum* genera. Among these, *T. tonsurans* is the most frequent cause of tinea capitis in the USA and urban communities in Western Europe [10,11,12], while *T. violaceum, T*. *soudanense,* and *M. audouinii* are mainly encountered in Africa [13]. Immigration and general movement of populations are responsible for a surge in infections in western countries due to species such as *T. soudanense* and *M. audouinii* [7,14].

Correct identification of the infection agent is essential in the determination of infection source, epidemiological course, and treatment initiation [15]. Conventional identification, which comprises the examination of macroscopic and microscopic features of in vitro cultures of clinical isolates, was until recently the main identification method for dermatophytes. This method is time-consuming, takes up to 4 weeks before distinct species characteristics are noticed, and requires experienced and well-trained staff [15,16,17]. The gold standard for dermatophyte identification is DNA sequencing, but it too is time-consuming and expensive for routine identifications [15,18]. In more recent years, matrix-assisted laser desorption ionization time-of-flight mass spectrometry (MALDI-TOF MS) has gained new ground in laboratories for routine identification of filamentous fungi such as dermatophytes. Each fungal species has its own unique protein profile, which is compared to a database with reference spectra of these profiles [19,20,21]. The main advantages of MALDI-TOF MS are its rapid and accurate identifications, relatively low cost, and easy integration into routine laboratory practice [18,21]. Its accuracy heavily depends on the quality of the reference spectra database [21].

Identification of clinical isolates with MALDI-TOF MS protocol requires a sub-culturing step for reliable identification. It can take days to weeks before fungal growth appears on solid medium [7,22,23]. In this study, a unique user-friendly and straightforward MALDI-TOF MS protocol using liquid cultures of dermatophyte species was developed in order to shorten the turnaround time for the culture and identification of clinical isolates. This protocol was first validated with reference strains and later applied to clinical isolates from *tinea capitis* patients.

## 2. Material and Methods

### 2.1. Study and Site Design

This study was conducted in the department of Mycology and Aerobiology, Sciensano, Brussels, Belgium. Clinical isolates from all suspected tinea capitis infections at the dermatology department from the University Hospital Brugmann, Saint-Pierre & Children Hospital Queen Fabiola (Brussels, Belgium) were collected between January 2019 and June 2019. All the samples were first analyzed in the mycology laboratory in the dermatology department through morphological identification, and afterwards sent to the Sciensano laboratory of Mycology and Aerobiology. When discrepancies occurred between identifications by the conventional method and MALDI-TOF MS, the identity of the species was determined using DNA sequencing.

### 2.2. Microorganisms

#### 2.2.1. Reference Strains

Reference strains of dermatophyte species were provided by the BCCM/IHEM fungal culture collection (Sciensano, Brussels, Belgium). Ten reference strains of the most common causative agents of ringworm were selected to validate the developed protocol (Table 1). Their identity was confirmed via DNA sequencing.

#### 2.2.2. Clinical Isolates

Sixty-one samples were collected from *tinea capitis* patients, consisting of scalp swabs and skin fragments. 

### 2.3. Subculturing Protocol

Reference strain: The strains were first inoculated on Petri dishes containing solid Sabouraud–chloramphenicol (SCh) medium. Next, liquid and solid sub-cultures were acquired by transferring fungal material to liquid and solid SCh, respectively. The liquid sub-cultures were incubated at room temperature (19–21 °C) on a rolling plate (30 rpm), while the solid sub-cultures were placed in an incubator at 25 °C. Strains were grown for 2 to 14 days prior to MALDI-TOF MS analysis.

Clinical isolates: Thirty-nine clinical isolates were inoculated on solid medium in the mycology laboratory of the dermatology department. In the Sciensano laboratory, they were sub-cultured on solid medium (Sch) (incubation at 25 °C) and in tubes with liquid Sabouraud–chloramphenicol (SCh) medium. The tubes were placed on a rolling plate and incubated at room temperature (19–21 °C). Liquid and solid sub-cultures were each incubated for 2 to maximum 14 days.

Twenty-two clinical isolates were directly placed in or onto the liquid/solid medium. These samples were initially inoculated in liquid and solid SCh medium. The liquid cultures were placed on a rolling plate and incubated at room temperature, while the solid cultures were incubated at 25 °C. Since a high level of contamination was observed using this method, the medium was switched to Sabouraud–chloramphenicol amended with actidione (cycloheximide) (SAC), to allow for a more specific selection of dermatophytes [24]. MALDI-TOF MS analysis was performed when the cultures had grown sufficiently, between 2 and 14 days of incubation, and also at fixed timepoints, i.e., after 3, 7, 14, and 21 days of incubation, to observe whether a protein spectrum could be detected even when no visible fungal growth could yet be seen.

### 2.4. The Liquid Media MALDI-TOF MS Protocol

MALDI-TOF MS protocol (using 1:3 volume of sterile water and anhydrous alcohol) described by Cassagne et al. (2011) was adapted for liquid instead of solid cultures [25]. Instead of scraping the fungal material from the surface of a solid culture, 1 mL of liquid culture (on Sabouraud–chloramphenicol medium amended with actidione (SAC)) was pipetted in a 1.5 mL Eppendorf tube. The cells were subsequently centrifuged for 10 min at 13,000 revolutions per minute (rpm). After discarding the supernatant, the remaining pellet was washed with 300 µL of sterile water and centrifuged for 10 min at 13,000 rpm. This washing step was performed twice. Afterwards, the pellet was dried completely and from thereon, the remaining steps were similar to the standard protocol by Cassagne et al. (2011) [25]. First, the pellet was re-suspended in 50 µL of 70% formic acid (Sigma-Aldrich, City of Saint Louis, MO, USA), vortexed and incubated for at least 5 min at room temperature. Then, 50 µL of pure acetonitrile (Sigma-Aldrich) was added to the pellet, again followed by vortexing and incubation at room temperature for 5 min. The mixture was subsequently centrifuged for 2 min at 13,000 rpm and 1 µL of the supernatant was pipetted onto four spots of a MALDI 96 polished steel target plate (Bruker Daltonics, Bremen, Germany). After complete drying, the spots were covered with 1 µL of α-cyano-4-hydroxycinnamic acid (HCCA) matrix solution (in 50% acetonitrile, 47.5% water, and 2.5% trifluoroacetic acid). The spots were dried completely at room temperature and analyzed using MALDI-TOF MS on default settings. Spectra of the spots were recorded in the positive linear mode in a mass range from 2–20 kDa. For each isolate, the spectra of the spots were compared to spectra of the BCCM in-house reference library. The MALDI BioTyper 3.0 software was applied (Bruker Daltonics, Bremen, Germany) to analyze the obtained spectra; instrument calibration was performed with BTS (bacterial test standard, Bruker Daltonics, Bremen, Germany). The MS-based identification of the tested dermatophyte species was considered acceptable if the mean of the best-match log scores was ≥1.70 for at least three out of four spots.

### 2.5. The Solid MALDI-TOF MS Protocol

To evaluate this newly developed liquid media MALDI-TOF MS protocol, it was compared to the standard protocol by Cassagne et al. based on solid cultures (2011) [25]. Following this protocol, fungal material was scraped from the surface of solid cultures and transferred into 300 µL of sterile water. Subsequently, 900 µL of absolute ethanol was added and the mixture was centrifuged for 10 min at 13,000 rpm. The supernatant was discarded and the pellet was air dried for at least 30 min to ensure no water or ethanol was left in the tube. Next, the cells were re-suspended in 50 µL of 70% formic acid, then vortexed and incubated for 5 min at room temperature. After incubation, 50 µL of pure acetonitrile was added and the suspension was vortexed again and incubated for 5 min at room temperature. The mixture was finally centrifuged for 2 min at 13,000 rpm, and 1 µL of the supernatant was placed onto the spots of the MALDI-TOF MS steel target plate. After air-drying, 1 µL of HCCA was applied to cover the spots and the spots were dried completely at room temperature. Spectra were recorded and analyzed in a similar manner to the liquid cultures.

### 2.6. Databases

In addition to the BCCM/IHEM in-house library, two other databases were used for the identification of the primary clinical isolates, with the purpose of examining whether different rates of correct identifications (RCI, in %) could be obtained. Spectra of the clinical isolates were compared to those of the filamentous fungi database 3.0 provided by Bruker (Bruker Daltoniks, Bremen, Germany) and the MSI V2.0 database provided by Assistance Publique Hopitaux de Paris (Paris, France), Sorbonne Université (Paris, France) and the BCCM/IHEM/Sciensano collection (Brussels, Belgium). The Bruker database is based on reference strains sub-cultured in liquid cultures. Identification using the Bruker database was identical to the in-house database: at least three out of four spots needed to have a mean of the best-match log score of ≥1.70 for the identification to be considered correct. In the case of the MSI database, the obtained identification score was required to exceed the threshold value of 20. Only the spot with the highest identification score was taken into account (Normand et al., 2017). This database and the BCCM/IHEM in-house library are based on solid cultures of reference strains.

### 2.7. Rate of Correct Identification and Turnaround Time

For each species of reference strain and the clinical isolates, the RCI was calculated, representing the percentage of correct identifications of isolates at the species level for that species. For the clinical samples, the turnaround time of the newly developed liquid media MALDI-TOF MS protocol was determined. The turnaround time was the incubation time of the isolates starting from the inoculation on solid or liquid medium until successful application of MS.

### 2.8. Spectra

The recorded spectra of the reference strains and clinical isolates were visualized and compared using the FlexAnalysis software, version 3.4 (Bruker Daltoniks, Bremen, Germany). The protein profiles of the isolates were compared by visualizing the signal intensity of the ions (in absorption units) as a function of their mass-to-charge ratio (*m*/*z*, in kg/C).

### 2.9. Blanks

In addition to the reference strains and clinical samples, blank samples of liquid and solid SAC were also analyzed with MALDI-TOF MS, to evaluate potential inference from spectra originating from the culture medium itself. These blank cultures were incubated at the same conditions as the clinical isolates, and were analyzed at 3, 7, 14, and 21 days of incubation. The solid and liquid samples were obtained by following the same protocols used for the clinical isolates.

## 3. Results

### 3.1. Validation of the Liquid Media MALDI-TOF MS Protocol (on BCCM/IHEM in-House Library)

Reference strains of ten dermatophyte species that commonly cause *tinea capitis* were used for validation of the liquid medium MALDI-TOF MS protocol, and a parallel with the MALDI-TOF MS protocol based on solid cultures was drawn (Table 1).

All reference strains were correctly identified with the liquid media MALDI-TOF MS protocol, except for *T. tonsurans*, which was misidentified as *T. interdigitale*, even when a retest was performed. The mean log scores of *T. soudanense* and *T. violaceum* strains were lower than 2.00, but were above 2.00 for all other strains. Overall, the MALDI-TOF MS protocol based on solid cultures led to a significantly higher mean log score for the identification of dermatophyte reference strains compared with the protocol based on liquid cultures, with respective average scores of 2.35 versus 2.14 (*p* = 0.024 on a 5% significance level).

### 3.2. Testing the Liquid Media Protocol on Sub-Cultures of Clinical Isolates (on BCCM/IHEM in-House Library)

The tested dermatophyte isolates were cultured in the mycology laboratory in the dermatology department of the University Hospital Brugmann, Saint-Pierre and Queen Fabiola Children’s Hospital (Brussels, Belgium) and afterwards inoculated in liquid and solid Sabouraud–chloramphenicol (SCh) medium in the laboratory of the Mycology and Aerobiology department of Sciensano. The number of samples for each species can be found in Table 2.

Using the liquid media MALDI-TOF MS protocol, a global rate of 62% correct identification (RCI) was obtained, compared with 87% for the protocol based on solid cultures (Table 3). Isolates that were not correctly identified with the liquid media protocol were mostly misidentified as other species (87%), while for the remaining 13% of isolates, reliable identification at species level could not be achieved.

Eighty-seven percent of the isolates of the *Trichophyton* genus were not correctly identified. *T. tonsurans* was not correctly identified in any of its isolates using liquid cultures, with 88% of the isolates misidentifying it as *T. interdigitale*, while an RCI of 88% was obtained for the solid cultures. For the *Microsporum* species, the rate of correct identification using the solid and liquid cultures was similar. Liquid cultures of *M. canis* isolates were all correctly identified.

### 3.3. Testing the Liquid Media Protocol on Primary Clinical Isolates (on BCCM/IHEM in-House Library)

The MALDI-TOF MS protocol based on liquid cultures was tested on a total of 22 clinical samples that led to dermatophytes isolation (Table 4). Eighteen of the clinical samples comprised swabs from the scalp, four samples consisted of skin flakes.

Overall, 41% of the primary clinical isolates were correctly identified at species level using the liquid protocol, in comparison with 90% using the solid protocol (Table 5). This percentage was lower than the RCI for the sub-cultures of clinical isolates (62%). Like the sub-cultures, difficulties with identifying *Trichophyton* species were encountered in the primary isolates, but correct identification of species from the *Microsporum* genus also decreased from 92% to 60%. Of the isolates on liquid medium that were not correctly identified, 69% could not be identified reliably at species level, while 31% were misidentified as other species. Of these misidentified species, 50% were *T. tonsurans* misidentified as *T. interdigitale* and 50% were *T. soudanense* misidentified as *T. rubrum*.

### 3.4. Other Databases

Two other databases were used for identification of the sub-cultured and primary dermatophyte isolates, to test whether a different database could lead to a higher RCI.

### 3.5. Bruker Database

The Bruker database is based on reference spectra from liquid cultures. The latest version of this database contained spectra of neither *M. audouinii* nor *T. soudanense*, which resulted in an RCI of 0% for these species (Table 3). Overall, RCI-values were lower using the Bruker database in comparison with the BCCM/IHEM in-house library, especially for primary isolates. Liquid cultures of *T. tonsurans* were correctly identified 63% of the time when a sub-culturing step was performed, but again there were no correct identifications for the primary isolates.

For primary isolates, RCI values remained low, with 40% and 20% for sub-cultured and primary isolates, respectively.

### 3.6. MSI Database

The MSI database is a library based on spectra originating from solid cultures. Using this database, 47% and 45% of liquid cultures were correctly identified for sub-cultured and primary isolates, respectively, representing higher RCIs for primary isolates than were obtained with the in-house database (Table 3). It was also noted that species of the *Microsporum* genus were more often correctly identified than species of the *Trichophyton* genus, which was in line with the results of the in-house database. Isolates of *T. soudanense* could not be correctly identified using the MSI database.

### 3.7. Turnaround Time

The turnaround times of the solid and liquid MALDI-TOF MS protocol were compared. For the sub-cultured clinical dermatophyte isolates, the turnaround time was 7.2 days with the solid protocol and 5.7 days with the liquid protocol. This was a significant difference (*p* = 0.005), but it is unknown how long the isolates had been in the hospital before being send to Sciensano for sub-culturing. For the primary isolates, the turnaround time was measured from direct inoculation onto the medium until MALDI-TOF MS analysis. Turnaround times of 11.7 and 11.6 days were calculated for the solid and liquid protocols, respectively, with no significant difference between the two methods (*p* = 0.96)

### 3.8. Performing the Liquid MALDI-TOF MS Protocol at Fixed Points in Time

For the primary isolates, the liquid MALDI-TOF MS protocol was performed at fixed timepoints (3, 7, 14, and 21 days after inoculation on SAC medium) to investigate whether spectra could be detected even when no visible fungal growth was seen, as most isolates began to show fungal growth only after 7 days of incubation. For all liquid isolates without visible fungal growth, no signal could be detected by MALDI-TOF MS, and no spectra were obtained.

### 3.9. Spectra

**Figure 1:**
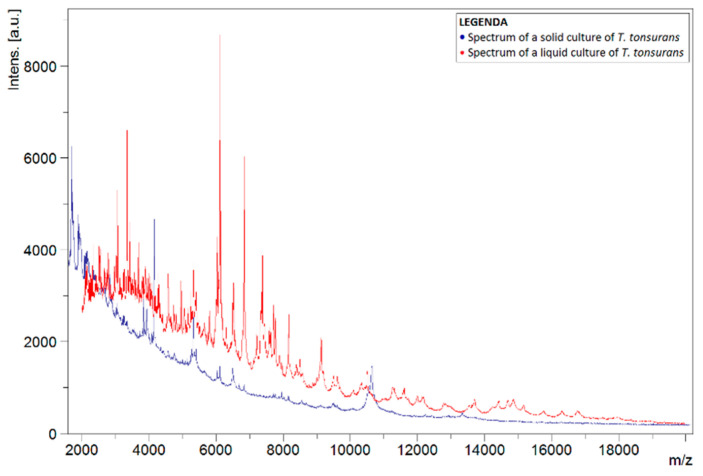
Spectra of liquid *T. tonsurans* isolate (red) and a solid *T. tonsurans* reference strain from the BCCM/IHEM collection (blue). Spectra show the signal intensity (Intens., in absorption units) of the ions detected by MALDI-TOF MS, as a function of their mass-to-charge ratio (*m*/*z*, in kg/C).

**Figure 2:**
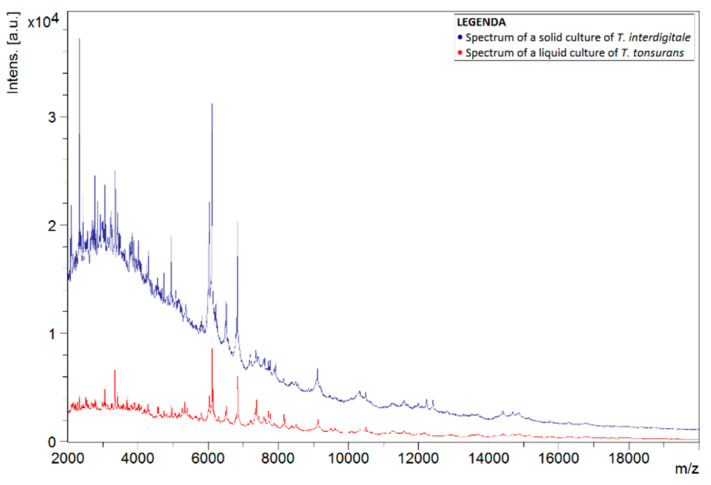
Spectra of liquid *T. tonsurans* isolate (red) and a solid *T. interdigitale* reference strain from the BCCM/IHEM collection (blue). Spectra show the signal intensity (Intens., in absorption units) of the ions detected by MALDI-TOF MS, as a function of their mass-to-charge ratio (*m*/*z*, in kg/C).

To investigate why the number of correct identifications using the liquid protocol was so low, the spectra of liquid and solid cultures of the same species were compared. This comparison was carried out for spectra of *T. tonsurans*, as this species was often misidentified as *T. interdigitale*. Figure 1 shows the spectra of liquid and solid isolates of *T. tonsurans*, in which differences between both spectra can noticed. Peaks were not concurrent for the most part. Comparison with a spectrum of a solid culture of *T. interdigitale*, found in the in-house database, showed greater concurrency in the peaks (Figure 2).

### 3.10. Blank Samples

**Figure 3:**
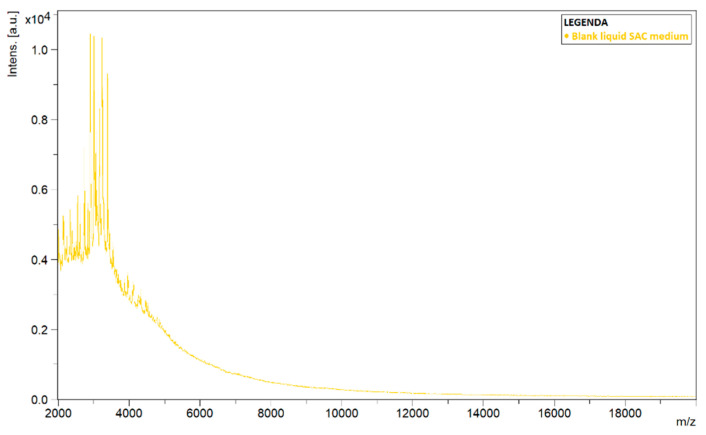
Detected spectrum of a blank sample of liquid Sabouraud–chloramphenicol medium amended with actidione (SAC). Spectrum shows the signal intensity (Intens., in absorption units) of the ions detected by MALDI-TOF MS, as a function of their mass-to-charge ratio (*m*/*z*, in kg/C).

To explain the differences in the spectra of liquid and solid cultures, blank SAC samples, i.e., medium without inoculation of a pathogen, were analyzed using MALDI-TOF MS. During analysis, it was noted that spectra continued to be detected in the liquid samples (Figure 3). The plotted spectrum shows that peaks of high intensity values could be observed in the lower mass-to-charge ratio (*m*/*z*) range. The signal died out with higher *m*/*z*-values.

## 4. Discussion

### 4.1. Evaluating the Liquid Protocol

The newly designed liquid MALDI-TOF MS protocol did not lead to a significantly shorter turnaround time for the identification of dermatophytes isolated from *tinea capitis* infections. When no visible fungal growth was present in the tubes with liquid medium, no signal could be detected by MALDI-TOF MS, and because it took as much time to grow the liquid cultures as the solid cultures, a faster identification could not be obtained. Moreover, the overall number of correct identifications reduced significantly when the method was compared to the standard protocol described by Cassagne et al. (2011) [25], especially when primary isolates of the samples were used. A sub-culturing step seems necessary to obtain reliable identifications; directly inoculating samples does not seem a useful tool to reduce turnaround time. The remarkably low rate of correct identification for *Trichophyton* species is also noteworthy. One hypothesis could be that some species grow more easily on liquid medium. Indeed, species such as *M. canis* that more often correctly identified with high RCI in general present fluffy colonies. In contrast, *Trichophyton* species such as *T. soudanense* and *T. violaceum*, as well as *M. audouinii*, generally present colonies that adhere better to solid medium. *T. tonsurans* strains were often misidentified as *T. interdigitale.* These species are genetically fairly similar (they are part of the same *T. mentagrophytes* series defined by de Hoog et al. using the ITS region as a marker), but they are well-distinguished species [2]. Sacheli et al. [26] encountered the same problem of cross identification between *T. tonsurans* and *T. interdigitale*, as did Hedayati et al. [27], presumably because the major peaks in the spectra of *T. tonsurans* are also present in those of *T. interdigitale* [21].

### 4.2. Evaluating the Bruker and MSI Databases

Using other databases did not lead to higher rates of correct identification. The Bruker 3.0 filamentous fungi database lacks the reference spectra of important causal agents of *tinea capitis,* such as *M. audouinii* and *T. soudanense*, which resulted in low rates of correct identification for solid and liquid dermatophyte cultures. When eliminating the data from the absent species, the overall RCI for both solid and liquid cultures remained unacceptably low for primary isolates, but those of the sub-cultured isolates increased to 78% and 83%, respectively. The Bruker database contains reference spectra originating from liquid cultures, which is presumably why more correct identifications could be obtained. Unlike the Bruker database, the MSI 2.0 database is based on mass spectra of solid cultures and contains spectra of all tested species. Using this database did not lead to an improvement in identifications, compared with the in-house database and the Bruker database. Here, it should also be noticed that identifying the *Trichophyton* species seems to be the biggest challenge, especially *T. soudanense.* This species is included in the database, but its isolates were never correctly identified in our study, even for the solid cultures.

### 4.3. Importance of the Database

Comparing the spectra of the liquid and solid isolates of the same species, it was clear that these spectra differed in intensity and peaks. It appeared that the liquid medium interfered with the detected MALDI-TOF MS signal, although several washing steps were performed on the pellets obtained from the liquid isolates. This interference was mostly noticeable at lower *m*/*z*-values, leading to higher intensities compared with the spectra of solid cultures. Extra washing steps should be added to the protocol, but this will increase the turnaround time of the method. Furthermore, it is known that strains grow differently in different types of growth media [28], so it could be that a strain grows differently in liquid or solid growth media, which could lead to a different protein pattern. Protein expression has a particular impact on the MALDI-TOF MS analysis; some authors showed that dark fungal pigments inhibited the desorption/ionization process during MALDI-MS [29]. Others authors reported that including species-specific spectra of young and mature colonies of the reference strains allowed identification regardless of the maturity of the clinical isolate [30].

The low RCI for the liquid protocol reflects the importance of a standardized protocol when using MALDI-TOF MS to identify fungi or microorganisms in general. It is necessary that cultures undergo the exact same steps as the reference strains in the database, from growth conditions such as temperature and growth medium to the handling of the obtained pellets. When using liquid cultures of clinical samples, the database used for identification by MALDI-TOF MS should also be based on liquid reference cultures. This theory was tested on *T. tonsurans*. Due to the high number of misidentifications as *T. interdigitale*, the *T. tonsurans* strains (spectra of reference strain, sub-cultured, and primary isolates) were also identified with MALDI-TOF MS using a small database based on liquid reference cultures (Table 5). This database included five *T. tonsurans* strains (confirmed by DNA sequencing) from the BCCM/IHEM collection (Table 6). The liquid cultures used for the database underwent the newly designed protocol described in the material and methods section, identical to the clinical isolates.

**Figure 4:**
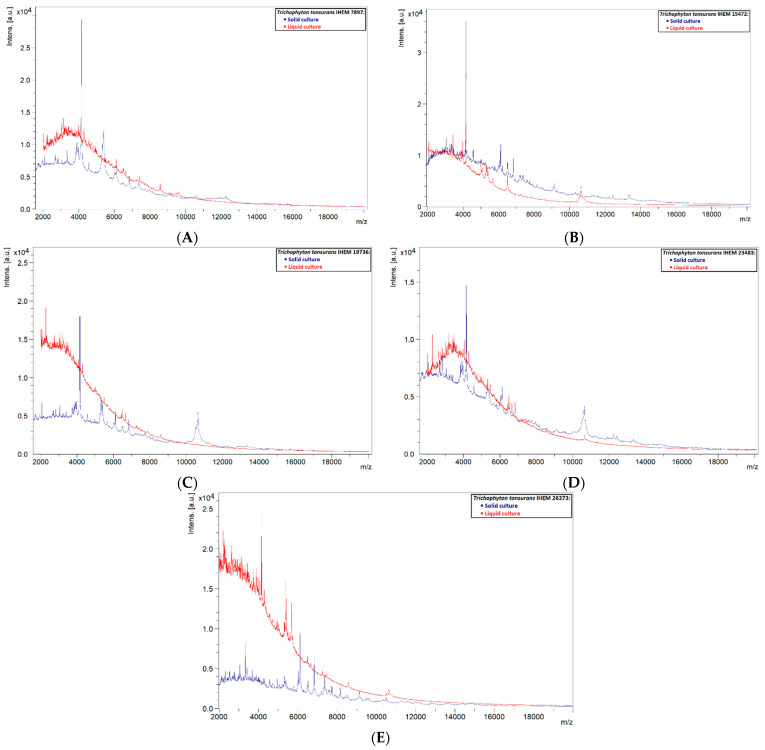
Comparison of solid and liquid cultures of the reference strains used in the liquid database. (**A**) IHEM 7897, (**B**) IHEM 15472, (**C**) IHEM 19736, (**D**) IHEM 23483 and (**E**) IHEM 26373.

Out of the 13 tested isolates, only one (primary isolate 21OS/144, from CHU Saint-Pierre) with an average score of 2.32 was reliably identified as *T. tonsurans*. The remaining 12 isolates had scores below 1.70, making the identifications unreliable. This showed that a liquid database may not be a good alternative for solid databases, and it might be suspected that spectra of liquid cultures are not as stable as those of solid ones. Spectra of solid and liquid cultures of the same strain were compared, and it was observed that in most cases peaks were less present or were not as clearly defined in the spectra of liquid cultures, with intensities often lower and peaks sometimes completely absent (Figure 4). Spectra of solid cultures contained well-defined peaks with high intensities, therefore it can be recommended to use a protocol based on solid cultures when applying the MALDI-TOF MS technique for identification of dermatophytes.

**Figure 5:**
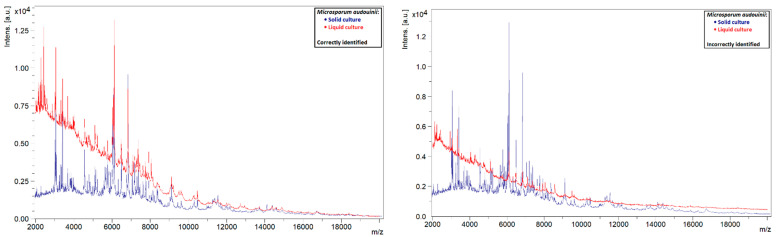
Comparison of the spectra of solid and liquid cultures of *M. audouinii*, on the left a correctly identified liquid culture and on the right an incorrectly identified liquid culture.

**Figure 6:**
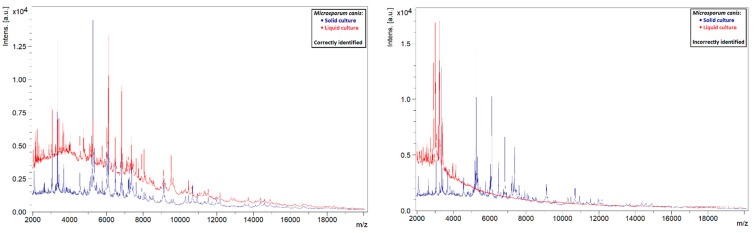
Comparison of the spectra of solid and liquid cultures of *M. canis*, on the left a correctly identified liquid culture and on the right an incorrectly identified liquid culture.

**Figure 7:**
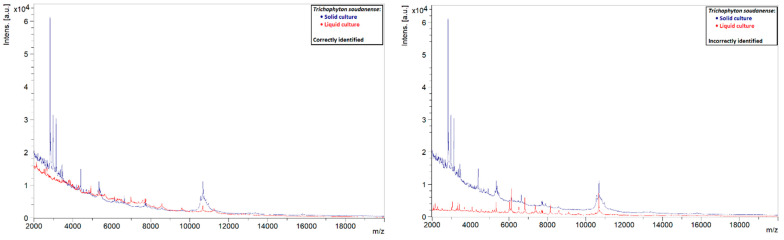
Comparison of the spectra of solid and liquid cultures of a *T. soudanense* strain, on the left a correctly identified liquid culture and on the right an incorrectly identified liquid culture.

**Figure 8:**
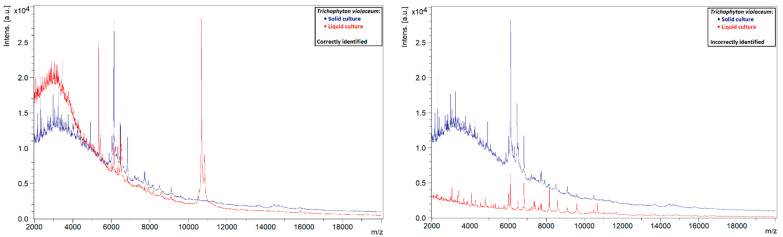
Comparison of the spectra of solid and liquid cultures of a *T. violaceum* strain, on the left a correctly identified liquid culture and on the right an incorrectly identified liquid culture.

A similar phenomenon was also observed in other species. Liquid strains that were correctly identified by MALDI-TOF MS showed clearly defined peaks with high intensities, while those that could not be identified showed rather flat spectra (Figure 5, Figure 6, Figure 7 and Figure 8). This indicates that the spectra of liquid cultures can differ strongly from each other, even when the same MALDI-TOF MS protocol is followed, which makes them harder to identify or to use as reference strains in databases.

### 4.4. Future Perspectives

Authors have reported that the results in the literature are independent from culture medium, incubation time, and MS instruments used by laboratories, but are heavily dependent on the quality of the reference spectra database [21]. In our study, we showed that the medium had an important impact on the MALDI-TOF MS analysis. This research has shown that even when eliminating the sub-culturing step and making use of primary isolates, no significant difference in turnaround time was attained using this newly developed liquid MALDI-TOF MS protocol. Overall, RCI values did not increase even when using a database based on liquid cultures. Although the number of isolates used in this experiment was small, it appears that sufficient valuable information was gathered, and a repeat of the experiment is not necessary.

As shortening the turnaround time is critical in clinical environment for rapid and correct treatment of patients, it could be effective to use ID-Fungi plates Plus medium (IDFPC) from Conidia^®^, specially developed for the direct inoculation of nails, hair and skin samples, and containing cycloheximide. Research by Sacheli et al. showed that the average time for a positive culture (sufficient fungal growth to perform MALDI-TOF MS analysis) was 6 days [26]. Isolates are easier to manipulate when grown on IDFPC, but further research is needed to obtain the RCI values of the solid Sabouraud medium. A database based on reference strains grown on IDFPC will presumably be necessary to obtain the most accurate identifications.

## 5. Conclusions

The newly developed liquid MALDI-TOF MS protocol has been proven an unsuitable alternative to the standard protocol for fast and accurate identification of clinical isolates of dermatophytes. The turnaround time of the method for primary isolates was not significantly lower and the rate of correct identification decreased tremendously, which emphasizes the need for a sub-culturing step. Using the Bruker 3.0 filamentous fungi database or the MRI 2.0 database did not lead to improvement in turnaround time or RCI. This study highlights the importance of the reference database when performing MALDI-TOF MS.

## Figures and Tables

**Table 1:** Validation of the liquid media MALDI-TOF MS protocol using BCCM/IHEM reference strains. The mean log score of all four spots (LS) of the liquid (LC) and solid cultures (SC) are listed, as well as their identity at species level (ID).

IHEM Reference Strains	MALDI-TOF MS SC		MALDI-TOF MS LC	
	ID	LS	ID	LS
IHEM 25452 *E. floccosum*	*E. floccosum*	2.15	*E. floccosum*	2.05
IHEM 26878 *M. audouinii*	*M. audouinii*	2.19	*M. audouinii*	2.34
IHEM 27271 *M. canis*	*M. canis*	2.27	*M. canis*	2.18
*IHEM* 26589 *N. gypsea*	*N. gypsea*	2.46	*N. gypsea*	2.34
IHEM 2771 *T. interdigitale*	*T. interdigitale*	2.55	*T. interdigitale*	2.40
IHEM 4270 *T. mentagrophytes*	*T. mentagrophytes*	2.18	*T. mentagrophytes*	2.33
IHEM 13886 *T. rubrum*	*T. rubrum*	2.49	*T. rubrum*	2.08
IHEM 27691 *T. soudanense*	*T. soudanense*	2.40	*T. soudanense*	1.81
IHEM *T. tonsurans*	*T. tonsurans*	2.36	*T. interdigitale*	2.28
IHEM 26519 *T. violaceum*	*T. violaceum*	2.08	*T. violaceum*	1.82
Overall average		2.35 ± 0.03		2.14 ± 0.04

**Table 2:** Number of samples of the sub-cultured clinical dermatophyte isolates per species.

Species	Number of Samples for Sub-Cultured Clinical Isolates
*M. audouinii*	8
*M. canis*	16
*T. tonsurans*	8
*T. soudanense*	6
*T. violaceum*	1
Total	39

**Table 3:** Rate of correct identification (RCI) per species, per genus, and overall, using the MALDI-TOF MS protocol based on solid (SC) or liquid cultures (LC).

	BCCM/IHEM in-House Library				Bruker Library				MSI Library			
	Sub-Cultures of Clinical Dermatophyte Isolates		Primary Dermatophyte Isolates		Sub-Cultures of Clinical Dermatophyte Isolates		Primary Dermatophyte Isolates		Sub-Cultures of Clinical Dermatophyte Isolates		Primary Dermatophyte Isolates	
**Species**	**RCI SC (%)**	**RCI LC (%)**	**RCI SC (%)**	**RCI LC (%)**	**RCI SC (%)**	**RCI LC (%)**	**RCI SC (%)**	**RCI LC (%)**	**RCI SC (%)**	**RCI LC (%)**	**RCI SC (%)**	**RCI LC (%)**
***Microsporum*** **sp.**	**88**	**92**	**100**	**60**	**61**	**61**	**0**	**10**	**83**	**57**	**100**	**70**
*Microsporum audouinii*	75	75	100	56	0	0	0	0	75	63	100	67
*Microsporum canis*	94	100	100	100	93	93	0	100	87	53	100	100
***Trichophyton*** **sp.**	**87**	**13**	**91**	**25**	**27**	**33**	**18**	**0**	**47**	**33**	**36**	**25**
*Trichophyton tonsurans*	88	0	75	0	50	63	50	0	75	63	100	75
*Trichophyton soudanense*	100	33	100	38	0	0	0	0	0	0	0	0
*Trichophyton violaceum*	0	0	/	/	0	0	/	/	100	0	/	/
**Clinical dermatophyte isolates overall**	**87**	**62**	**94**	**41**	**47**	**50**	**11**	**5**	**68**	**47**	**41**	**45**

The MBT Filamentous Fungi Library 3.0 version used during our study in 2019 contain the spectra of neither *M. audouinii* nor *T. soudanense*, resulting in an RCI of 0% for these species. When eliminating the species that were absent in the database, the overall RCI for sub-cultured isolates on solid and liquid media increased greatly, to respectively 78% and 83%.

**Table 4:** Numbers of clinical samples that led to dermatophytes isolation.

Species	Number of Samples for Primary Isolates
*M. audouinii*	9
*M. canis*	1
*T. tonsurans*	4
*T. soudanense*	8
Total	22

**Table 5:** Tested *Trichophyton tonsurans* isolates, using the liquid database.

Sample	Type	Species
IHEM26373	Reference strain	*Trichophyton tonsurans*
19/0009/27	Sub-culture	*Trichophyton tonsurans*
19/0009/46	Sub-culture	*Trichophyton tonsurans*
19/0009/52	Sub-culture	*Trichophyton tonsurans*
19/0009/53	Sub-culture	*Trichophyton tonsurans*
19/0009/60	Sub-culture	*Trichophyton tonsurans*
19/0009/73	Sub-culture	*Trichophyton tonsurans*
19/0009/82	Sub-culture	*Trichophyton tonsurans*
19/0009/83	Sub-culture	*Trichophyton tonsurans*
19/0009/14	Primary isolate	*Trichophyton tonsurans*
21OS/144	Primary isolate	*Trichophyton tonsurans*
21OS/145	Primary isolate	*Trichophyton tonsurans*
21OS/2284	Primary isolate	*Trichophyton tonsurans*

**Table 6:** Reference strains employed to set up a database based on liquid cultures, originating from the BCCM/IHEM fungal collection.

IHEM n°	Species
7897	*Trichophyton tonsurans*
15472	*Trichophyton tonsurans*
19736	*Trichophyton tonsurans*
23483	*Trichophyton tonsurans*
26373	*Trichophyton tonsurans*

## Data Availability

The data that support the findings of this study are available from the corresponding author, upon reasonable request.

## References

[1] Weitzman I., Summerbell R.C. (1995). The dermatophytes. Clin. Microbiol. Rev..

[2] de Hoog G.S., Dukik K., Monod M., Packeu A., Stubbe D., Hendrickx M., Kupsch C., Stielow J.B., Freeke J., Göker M. (2016). Toward a Novel Multilocus Phylogenetic Taxonomy for the Dermatophytes. Mycopathologia.

[3] Male O. (1989). Mycoses—A broad spectrum multi-faceted disease complex. Wien. Med. Wochenschr. 1946.

[4] Havlickova B., Czaika V.A., Friedrich M. (2008). Epidemiological trends in skin mycoses worldwide. Mycoses.

[5] Michaels B.D., Del Rosso J.Q. (2012). Tinea capitis in infants: Recognition, evaluation, and management suggestions. J. Clin. Aesthetic Dermatol..

[6] Leung A.K., Hon K.L., Leong K.F., Barankin B., Lam J.M. (2020). Tinea Capitis: An Updated Review. Recent Patents Inflamm. Allergy Drug Discov..

[7] Sacheli R., Harag S., Dehavay F., Evrard S., Rousseaux D., Adjetey A., Seidel L., Laffineur K., Lagrou K., Hayette M.-P. (2020). Belgian National Survey on Tinea Capitis: Epidemiological Considerations and Highlight of Terbinafine-Resistant T. mentagrophytes with a Mutation on SQLE Gene. J. Fungi.

[8] Elewski B.E. (2000). Tinea capitis: A current perspective. J. Am. Acad. Dermatol..

[9] Hay R.J. (2016). Tinea Capitis: Current Status. Mycopathologia.

[10] Fuller L.C., Barton R.C., Mohd Mustapa M.F., Proudfoot L.E., Punjabi S.P., Higgins E.M., Hughes J., Sahota A., Griffiths M., McDonagh A. (2014). British Association of D ermatologists’ guidelines for the management of tinea capitis 2014. Br. J. Dermatol..

[11] Abdel-Rahman S.M., Sugita T., González G.M., Ellis D., Arabatzis M., Vella-Zahra L., Viguié-Vallanet C., Hiruma M., Leeder J.S., Preuett B. (2010). Divergence among an International Population of Trichophyton tonsurans Isolates. Mycopathologia.

[12] Alshawa K., Lacroix C., Benderdouche M., Mingui A., Derouin F., Feuilhade de Chauvin M. (2011). Increasing incidence of Trichophyton tonsurans in Paris, France: A 15-year retrospective study. Br. J. Dermatol..

[13] Coulibaly O., L’Ollivier C., Piarroux R., Ranque S. (2018). Epidemiology of human dermatophytoses in Africa. Med. Mycol..

[14] Kieliger S., Glatz M., Cozzio A., Bosshard P. (2015). Tinea capitis and tinea faciei in the Zurich area-an 8-year survey of trends in the epidemiology and treatment patterns. J. Eur. Acad. Dermatol. Venereol..

[15] Gräser Y., Scott J., Summerbell R. (2008). The New Species Concept in Dermatophytes—A Polyphasic Approach. Mycopathologia.

[16] Kanbe T. (2008). Molecular Approaches in the Diagnosis of Dermatophytosis. Mycopathologia.

[17] Nenoff P., Erhard M., Simon J.C., Muylowa G.K., Herrmann J., Rataj W., Gräser Y. (2013). MALDI-TOF mass spectrometry—A rapid method for the identification of dermatophyte species. Med. Mycol..

[18] Packeu A., De Bel A., L’Ollivier C., Ranque S., Detandt M., Hendrickx M. (2014). Fast and Accurate Identification of Dermatophytes by Matrix-Assisted Laser Desorption Ionization–Time of Flight Mass Spectrometry: Validation in the Clinical Laboratory. J. Clin. Microbiol..

[19] Marvin L.F., Roberts M.A., Fay L.B. (2003). Matrix-assisted laser desorption/ionization time-of-flight mass spectrometry in clinical chemistry. Clin. Chim. Acta.

[20] Murray P.R. (2012). What Is New in Clinical Microbiology—Microbial Identification by MALDI-TOF Mass Spectrometry: A paper from the 2011 William Beaumont Hospital Symposium on molecular pathology. J. Mol. Diagn..

[21] L’Ollivier C., Ranque S. (2017). MALDI-TOF-Based Dermatophyte Identification. Mycopathologia.

[22] Erhard M., Hipler U.-C., Burmester A., Brakhage A.A., Wöstemeyer J. (2008). Identification of dermatophyte species causing onychomycosis and tinea pedis by MALDI-TOF mass spectrometry. Exp. Dermatol..

[23] Azrad M., Keness Y., Nitzan O., Pastukh N., Tkhawkho L., Freidus V., Peretz A. (2019). Cheap and rapid in-house method for direct identification of positive blood cultures by MALDI-TOF MS technology. BMC Infect. Dis..

[24] Rosenthal S.A., Furnari D. (1957). The Use of a Cycloheximide-Chloramphenicol Medium in Routine Culture for Fungi. J. Investig. Dermatol..

[25] Cassagne C., Ranque S., Normand A.-C., Fourquet P., Thiebault S., Planard C., Hendrickx M., Piarroux R. (2011). Mould Routine Identification in the Clinical Laboratory by Matrix-Assisted Laser Desorption Ionization Time-Of-Flight Mass Spectrometry. PLoS ONE.

[26] Sacheli R., Henri A., Seidel L., Ernst M., Darfouf R., Adjetey C., Schyns M., Marechal L., Meex C., Arrese J. (2020). Evaluation of the new Id-Fungi plates medium from Conidia for MALDI-TOF MS identification of filamentous fungi and comparison with conventional methods as identification tool for dermatophytes from nails, hair and skin samples. Mycoses.

[27] Hedayati M.T., Ansari S., Ahmadi B., Armaki M.T., Shokohi T., Taghizadeh Armaki M., Er H., Özhak B., Öğünç D., Ilkit M. (2019). Identification of clinical dermatophyte isolates obtained from Iran by matrix-assisted laser desorption/ionization time-offlight mass spectrometry. Curr. Med. Mycol..

[28] Meletiadis J., Meis J.F.G.M., Mouton J.W., Verweij P.E. (2001). Analysis of Growth Characteristics of Filamentous Fungi in Different Nutrient Media. J. Clin. Microbiol..

[29] Buskirk A.D., Hettick J.M., Chipinda I., Law B.F., Siegel P.D., Slaven J.E., Green B.J., Beezhold D.H. (2011). Fungal pigments inhibit the matrix-assisted laser desorption/ionization time-of-flight mass spectrometry analysis of darkly pigmented fungi. Anal. Biochem..

[30] Alanio A., Beretti J., Dauphin B., Mellado E., Quesne G., Lacroix C., Amara A., Berche P., Nassif X., Bougnoux M.-E. (2011). Matrix-assisted laser desorption ionization time-of-flight mass spectrometry for fast and accurate identification of clinically relevant Aspergillus species. Clin. Microbiol. Infect..

